# Premature Burial

**Published:** 1914-05-16

**Authors:** 


*To the Editor of* The Hospital.

### Premature Burial.

*To the Editor of* The Hospital.

Sir,—On reading Mr. Williamson's letter it strikes me that it is very necessary to frighten the easy-going "man in the street" into a practical realisation of the insecurity of the present Burial Laws to prevent live burial. When trance is more generally studied, and its subtle aping of death realised by the medical practitioner, perhaps then the medical faculty will bring their powerful influence to bear on the legislation for a reformed Burial Law which will be efficacious in preventing live burial in this country.—Yours, etc., E. L.

